# Immediate Effect of Kinesio Taping on Lumbopelvic Stability in Postpartum Women With Diastasis Recti: A Review

**DOI:** 10.7759/cureus.33347

**Published:** 2023-01-04

**Authors:** Yukti Jobanputtra, Shubhangi Patil

**Affiliations:** 1 Department of Physiotherapy, Ravi Nair Physiotherapy College, Datta Meghe Institute of Higher Education and Research, Wardha, IND; 2 Department Community Health Physiotherapy, Ravi Nair Physiotherapy College, Datta Meghe Institute of Higher Education and Research, Wardha, IND

**Keywords:** active straight leg raise, lumbo pelvic instability, kinesio taping, kinesio tape, diastasis recti

## Abstract

Diastasis of abdominal muscles is defined as a gap of more than two finger breadths between two rectal abdominal muscle bellies, above or below the umbilicus. According to the literature, female diastasis recti is more common in women who have recently given birth although it also occurs in the male population. Lower back pain is the most prevalent reason for postpartum women to restrict their everyday activities. Women's mobility, discomfort, and typical activities are all concerns for postpartum women, all of which change a person's quality of life. During pregnancy, diastasis of the rectus abdominal muscles is very common to occur. It is linked to instability of lumbar pelvic region and pelvic floor improper function. Recently, the use of Kinesio taping is gaining popularity as a technique to reduce the separation and increase stability. The review of studies revealed that Kinesio taping is highly effective for diastasis recti and in improving the stability of lumbo-pelvic spine which was evaluated by an active straight leg raise test. There are just a few studies accessible that show the need for further research.

## Introduction and background

Diastasis recti abdominis (DRA) is the excess separation of the rectus abdominis along with stretch and thinning of Linea alba (LA) that may occur due to any functional or mechanical disturbance in the walls of abdomen or the complete body [1]. A gap of more than 2 cm in between rectus abdominis muscle is considered as pathological and can occur in both men and women at any age [2]. Increased inter-recti distance compromises these processes and can lead to changes in biomechanics of trunk, stability of the pelvic joint, and alignment, making the lumbar and pelvis spine more prone to injury [3]. Occurrence of mild diastasis recti is seen more during pregnancy and the postpartum phase increasing age, obesity, caesarean section, multiple pregnancies, birth weight, are considered as risk factors for this this can also occur in infants, and people who have abdominal obesity and who are involved in jobs of lifting heavy objects [4]. The abdominal musculature plays a significant action in proper alignment of spine, stability of the back, proper breathing, and movements of the trunk, along with support to abdominal viscera [5]. This broadening has been linked to a variety of health problems, including lumbopelvic instability, low back discomfort, and incontinence [6]. Various estimates of inter recti distance incidence have been known, that ranges from 66% to 100% during around 28 weeks of pregnancy and goes till 53% in the postnatal period. Regular exercise prior to pregnancy and during the antenatal period appears to reduce the risk of diastasis recti development and reduce the distance [7]. Postnatal women with DRA are routinely offered abdominal workouts. Postural and appropriate care of back instruction, external support like tub grip and corset can be used, and cardiovascular exercise is all commonly utilized non-surgical therapies in such women [8]. Kinesio taping (KT) has become very popular in this case recently. Applying these tapes can prevent further stretching of LA and also act as supporting therapy for it [9]. The Active Straight Leg Raise (ASLR) Test, which includes to lift up and lower down a complete straight leg in a supine position simultaneously keeping the lumbar spine in a neutral position, is now a clinical method for assessment of lumbar pelvic region stability, abdominal muscle activation, and the severity of pelvic pain during and after pregnancy [10]. Our objective was to present a summary of relevant literature on the Immediate effect of KT on lumbopelvic stability in postpartum women with separation of rectus abdominis muscle.

## Review

Data sources and search engine

Original articles, systematic reviews, meta-analysis, and randomized control trials were among the studies chosen to include in this review. Keywords and the Medical Subject Headings (MESH) terms were used to find the articles. Screening of the articles was also done using keywords. For selecting the articles the keywords used were diastasis recti, KT, lumbopelvic instability, and active straight leg raise test. The online search engines PubMed, Scopus, Web of Science and Google Scholar were used to collect articles.

Methodology

There are on a total of 31 records of systematic literature after excluding six irrelated studies and 15 duplicates, 12 were assessed for eligibility that are full-text articles. Out of which total eight number of studies were included below in this review. The inabilities to access the full version of articles were among the main reasons for the exclusion. Hence there is a need for more literature regarding the same.

Diastasis recti abdominis

Diastasis recti abdominis (DRA) is a process marked by the gap between both the rectus abdominis muscles along the LA. However, alternative terminology, such as rectus abdominis diastasis (RAD) or rectus abdominal muscle divarication, are sometimes used in the literature [11]. Beer et al. in their study stated that the breadth of LA is 15 mm, at the upper umbilicus level it is 22 mm at 3 cm, and below umbilicus it is said to be 16 mm at 2 cm, in accordance to physiological criteria. With ageing, the LA expands in size [12]. A diastasis rectus abdominis of more than 25 mm can be considered harmful due to the influence of the abdominal muscles’ strength [13,14]. Different inter recti distance (IRD) measurement methods are used in clinical practice. Palpating, measuring through tape, calipers, USG, computerized tomography, and magnetic resonance imaging are some of the tools that can be used [15]. It was recommended that ultrasound and calipers are the suitable ways for assessing IRD based on the results of 13 research examining measuring characteristics [16]. Fernandes da Mota et al. [17] and Sperstad et al. [4] conclude that there was no link between diastasis and pre-pregnancy BMI, weight growth, weight of baby while birth or circumference of the belly, lifting heavy weights, and carrying infants, or daily exercise. According to the findings, DRA was found to be 68% above the umbilicus and 32% below the umbilicus in Brazilian research conducted shortly after vaginal delivery. Below umbilicus diastasis recti occurs more in multipara but occurs in same percent in multipara and primigravida above umbilicus [13]. The presence of DRA is linked to diagnoses of support-related pelvic floor dysfunction, according to the study [14]. The power of the abdominal musculature is influenced by an increase in the gapping between the rectus muscles, which normally causes no pain while rest. However, due to an increase in intraabdominal pressure, the distinctive bulge of the abdominal wall may emerge during vigorous activities. DRA may be linked to epigastric and umbilical hernias for this reason [18]. Because core muscle deficiency can cause trunk and pelvic instability, a change in pattern of breathing, over mobility in the lumbar pelvic region, and imbalance of posture, it's important to strengthen them [2]. In case-control research in 2020, the authors of the study focused on women suffering or not suffering from DRA after childbirth. The findings revealed that patients who had DRA had more problems with lumbar pelvic control and postural balance than fit people also, when compared to healthy people, these patients' lumbopelvic proprioception was dramatically diminished. The issues indicated might cause a shortage of muscular support on the lumbar spine, resulting in pain and incapacity in daily tasks [19]. The Active Straight Leg Raise (ASLR) Test is a diagnostic test used for evaluating instability of lumbar pelvic spine [10]. Participants perform to elevate one leg 20 cm (heel to plinth) while laying supine on a bed, hold there for five seconds, and then steadily return the leg back to the starting position on the bed. The spine and pelvis are tested for stability. On a scale of 0-5, participants rate the difficulty of elevating their leg (0=not at all difficult, 5=unable to lift the leg). The test would be redone with the examiner applying manual compression to the pelvis for ratings greater than zero. The test was said to be positive for pelvic instability if the difficulty level reduced with manual compression [20-21].

Kinesio tapes (KT)

KT was developed by Kenzo Kase, that is the combination of techniques of kinesiology and chiropractic that is based on different flexible and is based on distinct flexible strips that has same elasticity and density of natural skin. The strip is flexible longitudinally, whereas the wavy adhesive gives mechanical functioning of skin. No latex, chemical constitute, or medicine are constituted on the tape its made up of cotton fiber and has property of heat sensitive and resistant to the water. It is said to improve lymphatic drainage, train proprioception, reduce pain, reinforcing proper structure of motion, and decreasing imbalance of muscle [22]. This taping method is helpful for supporting joint functions by stabilizing muscles, improving functioning of the muscles, and endogenous analgesic mechanisms, along with enhancement in microcirculation. This technique is noninvasive type of physical therapy that include applying special tapes directly to the sin of affected area with a proper pattern. It is a proprioception that is known to be necessary in regulating tone and stability of muscles [23]. His technique has gained popularity in various musculoskeletal conditions treatment and can also be used during rehabilitation. It is more elastic and thinner compared to rigid band. Due to its air-permeable and water-resistant structure, this may be there sticked on the skin for around three days [24]. Taping joints improve not only mechanical joint stability directly, but it may also improve signals of various necessary areas like breathing, proper posture, continence ensurance, and bending of trunk are done by abdominal muscles [25]. Thus, it is necessary to treat it. According to the statistics every 24 out of 40 women needs rehabilitation for diastasis recti. However, the most known form of rehabilitation includes abdominal muscles strengthening which if performed in wrong pattern could cause the condition to worsen [26]. Most of the women overcome diastasis recti in phase of postpartum and in some condition where it remains same then can be treated by conservative technique, i.e., physiotherapy, which is said to be the only treatment method that can potentially reduce inter recti. Also, various studies confirms the positive outcome of exercises to decrease the gaping [26]. However, in recent years a technique known as KT that involves application of that tapes directly on the skin is becoming very popular In diastasis recti, applying this tapes can prevent further more stretching of Linea alba along with it has a supportive function [27].

Treatment

Taping technique supports self-healing property of the body. Physiotherapists are the ones who take decision for implementing such techniques in case of diastasis recti abdominis pattern of application, orientation, and tension of tape varies according to aim and the stage of rehabilitation, protection of Linea alba, preventing hernia, supporting regeneration of tissue and giving the strengthening effects of physiotherapy are the main aims for taping in diastasis recti [28]. It's made completely of cotton fiber and is water-resistant as well as temperature-sensitive. Taping is also said to improve lymphatic drainage as a effect of stimulated reactivation, proprioception training, pain reduction, reinforcing the appropriate structure of motion, and reducing imbalance of muscles [22]. According to the study done by Lucyna Ptaszkowska showed that KT tapes may be effective reducing diastasis recti which may further decrease its complication of lumbopelvic instability.

Discussion

The muscles such as diastasis are necessary to align posture, appropriate breathing, and movement of the trunk for which the main aim of using physiotherapy is to bring back the proper functions of the muscle that has become weak [13,25]. As such there’s no decided protocol for this, it depends upon the severity and results to various consequences [29-31]. Recently taping is a very known intervention of no-invasion physiotherapy that involves sticking the tapes direct on the skin with the correct way. It relies on the principle that helps the body to heal by itself. In case of separation of rectus abdominis muscle physiotherapist decides that it is to be implemented or not. This taping protects Linea alba, protect hernia, strengthen muscle, and provide stability [28].

After pregnancy, women recover from this at different periods of time. Typically postnatal phase is from 6-8 weeks during which this condition may be regained after pregnancy. It is found that during the second trimester it occurs 27% and 66% for third trimester pregnant women. However, in a study done by Mota et al. [25], it was said that it can occur in 100% also Brusch [2] found that out of 40 number of women 60% of them were requiring rehabilitation. Thabet and Alshehri [32] commented on an exercise program to stabilize the deep muscles of the core. In which he examined 40 women and divided into two groups the first group was said to perform abdominal and deep core exercise to strengthen it and the second group were said to perform only abdominal exercise this therapy has taken place for 8 weeks three session per week. It was found a statistically good reduce in decrease in the gaping in former. Kamel and Yousif [33] performed a study on 60 number of women and two months postpartum who were divided into two parts out of which first part did exercises of muscles of abdomen, and electrostimulation of the rectus abdominis and other part only did exercises for strengthening. A visible improvement (p < 0.05) in reduction of diastasis recti was seen in both groups, although previous part gained more of the advantage.

Benjamin et al. did an examination of the relationship between rectus muscle diastasis and lower area pain in the back, incontinence, and prolapse of the pelvic organ, as well as between abdominal power and quality of life [6]. Number of 2242 women with diastasis were included in the study but it had no find proper relation between the presence of separation and lumbar and pelvic pain or involuntary loss of urine. However, it demonstrated link between the presence of gap and prolapse.

Benjamin et al. [5] conducted a meta-analysis of the literature that is available in EMBASE, MEDLINE, CINAHL, PubMed, and PEDro. The main aim focussed to find out exercise to reduce gap between muscle. He overlook eight studies that included 336 pregnant and postnatal women. This method ranged from case studies to randomized control trials. It was found that before delivery if physical activity are focused then it reduces the risk of diastasis by 35%. The limitations found in most of the study was inclusion of few participants and a small span of time for tape application. The other disadvantage of study was subjective evaluation of the separation of the diastasis. Various trials were also done by Walton et al. [34], Bobowik and Dąbek [35], Keshwani et al. [36].

Ultrasonography would be a more meaningful test for the abdominal wall as it gives more precise measurement. The span of therapeutic effect of this technique remained unknown due to absence of regular follow-up. Hence, there is need for researches of techniques of physiotherapy and supportive therapy of RAD in postpartum women. Table 1 shows that various studies took place regarding diastasis and whether the treatment chose were helpful or not.

**Table 1:** Various studies regarding diastasis recti RCT: randomized clinical trial; IRD: inter-recti distance; KT: Kinesio taping; TRA: transverse recti abdominis; NMES: neuromuscular electrical stimulation

Author	Country	Study	Participants (Number, Age, Postpartum Period)	Delivery And Parity Mode	Rectus Abdominis Cut Off Value	Intervention and Outcome Measures	Secondary Outcome Measures	Result
Ptaszkowska et al, 2021 [1]	Poland	RCT	N=24, age 18-38, 6 weeks to 12 months after childbirth,	Multiple pregnancies, cesarean delivery were excluded	RAD > 2 cm at least at one of three sites	KT tapes, sham KT group non-stretch tapes were used as an intervention, and surface electromyography	Calipers, questionnare	Notable decrease in separation at each of the sites assessed after the use of KT in the treatment group (p < 0.05). In comparison, statistically lower RAD on umbilicus level was found after treatment. (p = 0.005) in KT group. This resulted that use of tapes as a corrective technique can result in decrease in RAD in women up to 12 months after delivery.
Walton et al., 2016 [34]	USA	RCT	N=9, 18-45 years, 3 months to 3 years postpartum	Parity was not reported. C- section and normal labor (n=1)	Not reported	using a caliper and ultrasound separation of muscle is measured at 4.5 cm below and above the umbilicus	Oswestry Disability Index, Pelvic Floor Distress Index	After treatment experiment and traditional: IRD in the umbilicus level, it was not noticed in any reduced IRD between both
Kamel and Yousif, 2017 [33]	Egypt	CT	N=60, 25-35 years, 2 months postnatal	Primiparous and multiparous. Normal delivery	During a curl up more than 2.5 cm measured any place along the Linea alba	Distance is calculated on ultrasound at midway between the navel and xiphoid process; midway between symphysis and navel	Abdominal muscle strength	Post-intervention: abdominal exercise + NMES: abdominal exercise: visible difference in reduction in gap between groups
Bobowik and Dąbek, 2018 [35]	Poland	RCT	N=40, age 32. ± 5.9 years postpartum time from 0 to 3 days	Pregnancy and delivery mode not reported	More than 2 cm	Measured with palpation assuming a finger width equal to 1.3 cm		Post-test: minimal intervention; physical therapy: significant difference present
Tuttle et al., 2018 [9]	USA	Pilot RCT	N=30, age 32.03 ±4.3 years, 6-12 weeks postpartum time	Primi and multiparous. Delivery mode not reported	More than 2-finger gap during head lift	Ultrasound is used to measure distance 4.5 cm below and above umbilicus, while head lifting and rest	Pelvic Floor Distress Index-20, the Roland- Morris Disability Questionnaire	Post-test TRA: IRD: 1.34 ± 0.37 minimal intervention: IRD: 2.1 ±0.99. Close to a significant difference in IRD between groups: significant better decrease in IRD at rest and during head lift in the groups with TRA training compared to control/tape
Gluppe et al., 2018 [27]	Norway	RCT	175 population was included. Age 29.8. Adding 4 years up or down years around 6 weeks postpartum	Primiparous, vaginal delivery	More than 2-finger widths or a visible protrusion during a curl-up	DRA measurement of diastasis with palpation during modified sit up movement 4.5 cm up and down of umbilicus 6- and 12 months postpartum		Post-test 6 months: Exercise:, 43.7%: 44.3% for 12 months: 41.4% minimal intervention: 39.8%. No visible difference between groups 6 months postpartum
Thabet and Alshehri, 2019 [32]	Saudi Arabia	RCT	N=40, 22-35 years, 3-6 months postpartum	Parity and delivery mode not reported	>2 cm from the umbilicus to 4.5 cm above the umbilicus or a visible protrusion	Distance measured with caliper 4.5 cm above umbilicus during modified sit-up	The Physical Functioning Scale	Training of deep core: IRD and traditional exercises; IRD: observable changes in IRD between groups. Hence former stability exercise program is effective
Keshwani et al., 2021 [36]	Canada	Pilot RCT	N=32, 31+3 years, 22 days postpartum	Primiparous, vaginal delivery	More than 2-finger gap at 2 cm above, 5 cm above, or 3 cm below umbilicus	IRD measured with ultrasound at umbilicus, 3 cm above, 5 cm above, or 3 cm below umbilicus	Abdominal muscle, Pelvic Floor Distress Index, body image, inventory of functional status after childbirth	Post-test: 6 months exercise therapy: IRD: 0.93 ± 0.88 abdominal binding: IRD: 1.34 ± 0.34; (combining: IRD: 1.24 ± 0.73). Minimal intervention: IRD: 1.31 ± 1.08. No noted differences. When comparing exercise therapy to control, no significant difference between groups was found

## Conclusions

This review concluded that the application of KT has proved effective to reduce diastasis recti in postpartum women that further would decrease instability of the lumbopelvic spine and increase the strength of the abdominal muscles which would enhance their quality of life. This intervention will reduce the separation between muscles and increase the stability of the lumbopelvic spine. It also suggests the need and requirement for more literature on the same.
